# NOVEL METHIONINE-RELATED INTERVENTIONS THAT CONFER HEALTHSPAN BENEFITS TO YEAST AND RODENTS

**DOI:** 10.1093/geroni/igz038.263

**Published:** 2019-11-08

**Authors:** Jay E Johnson, Jason D Plummer, Spike D Postnikoff, Jessica K Tyler, Jay E Johnson

**Affiliations:** 1 Orentreich Foundation for the Advancement of Science, Cold Spring, New York, United States; 2 Weill Cornell Medicine, New York, New York, United States

## Abstract

Methionine restriction (MR) is one of only a few dietary manipulations known to robustly extend healthspan in mammals. Methionine-restricted rodents are up to 45% longer-lived than control-fed littermates and a number of studies suggest that humans may also benefit from MR. While a methionine-restricted human diet is technically feasible, compliance to such a regimen might not be practical or desirable. Therefore, an important goal is to identify and/or develop more facile dietary interventions, or preferably, pharmacological agents that mimic MR. Towards this end, we have made use of the yeast chronological lifespan and replicative lifespan assays, which serve as models of aging in quiescent and mitotic cells, respectively. Importantly, our lab and others have demonstrated that MR dramatically extends yeast lifespan. Here we show work aimed at developing novel MR-like interventions that extend yeast lifespan, as well as preliminary data demonstrating that such interventions significantly improve the healthspan of mice.

